# Alternative migratory strategies related to life history differences in the Walleye (*Sander vitreus*)

**DOI:** 10.1186/s40462-022-00308-7

**Published:** 2022-03-02

**Authors:** Graydon McKee, Rachael L. Hornsby, Friedrich Fischer, Erin S. Dunlop, Robert Mackereth, Thomas C. Pratt, Michael Rennie

**Affiliations:** 1 Department of Biology, Lakehead University, Thunder Bay, ON P7B5E1 Canada; 2 Upper Great Lakes Management Unit, Ontario Ministry of Natural Resources and Forestry, Thunder Bay, ON P7E6S7 Canada; 3 Aquatic Research and Monitoring Section, Ontario Ministry of Natural Resources, Peterborough, ON K0L0G2 Canada; 4 Center for Northern Forest Ecosystem Research, Ontario Ministry of Natural Resources and Forestry, Thunder Bay, ON P7E2V6 Canada; 5 Great Lakes Laboratory for Fisheries and Aquatic Sciences, Fisheries and Oceans Canada, Sault Ste. Marie, ON P6A2E5 Canada; 6 International Institute for Sustainable Development Experimental Lakes Area, Winnipeg, MB R3B0Y4 Canada

**Keywords:** Multistate mark-resight, Thermal-Optical Habitat Area (TOHA), GLATOS, Sexual dimorphism, Pace of Life Syndrome (POLS), Telemetry, Laurentian Great Lakes

## Abstract

**Background:**

While Pace of Life Syndrome predicts behavioural differences between individuals with differential growth and survival, testing these predictions in nature is challenging due to difficulties with measuring individual behaviour in the field. However, recent advances in acoustic telemetry technology have facilitated measurements of individual behaviour at scales not previously possible in aquatic ecosystems.

**Methods:**

Using a Walleye (*Sander vitreus*) population inhabiting Black Bay, Lake Superior, we examine whether life history characteristics differ between more and less mobile individuals as predicted by Pace of Life Syndrome. We tracked the movement of 192 individuals from 2016 to 2019 using an acoustic telemetry study, relating patterns in annual migratory behaviour to individual growth, and seasonal changes in optimal thermal-optical habitat.

**Results:**

We observed two consistent movement patterns in our study population—migratory individuals left Black Bay during late summer to early fall before returning to the bay, whereas residents remained within the bay year-round. The average maximum length of migrant Walleye was 5.5 cm longer than residents, and the sex ratios of Walleye caught during fall surveys was increasingly female-biased towards the mouth of Black Bay, suggesting that a majority of migrants were females. Further, Walleye occupancy outside of Black Bay was positively associated with increasing thermal-optical habitat.

**Conclusions:**

Walleye in Black Bay appear to conform to Pace of Life Syndrome, with migrant individuals gaining increased fitness through increased maximum size, which, given size-dependent fecundity in this species, likely results in greater reproductive success (via greater egg deposition vs. non-migrants). Further, apparent environmental (thermal) controls on migration suggest that migratory Walleye (more so than residents) may be more sensitive to changing environmental conditions (e.g., warming climate) than residents.

**Supplementary Information:**

The online version contains supplementary material available at 10.1186/s40462-022-00308-7.

## Background

The theoretical concept of the Pace of Life Syndrome (POLS) provides a framework for understanding how animal behaviour relates to individual life history variation [37]. Life history variability results in differences in age-structured productivity and mortality, with bold and highly mobile individuals expected to have greater food intake, growth rates, and reproductive output than more cautious individuals [4]. These advantages of bold behaviour, however, come with trade-offs of higher energetic costs and predation risk [36, 37]. While ecosystems with scarce or abundant resources often drive evolution to highly mobile or sedentary strategies respectively, unstable or intermediately favourable conditions are well suited to behavioural variability in movement patterns [29]. Under such conditions, the POLS predicts that individuals will vary in a suite of behavioural and physiological characteristics associated with life history [36, 37].

Continually shifting environmental conditions can lead to challenges in identifying behavioural patterns and individual personalities, highlighting the importance of long term study [31, 36]. Disparate movement behaviours have been observed in spatial ecology studies with links to other personality features [12, 17], and in some cases have identified links between movement patterns with life history [13]. Documenting movement patterns in natural environments has historically proved challenging without intensive time and monetary investments, but advancements in technology are making such studies increasingly more feasible, particularly for fishes [11, 28].

The emerging field of passive acoustic telemetry, which makes use of hydrophone-receiver arrays to record locations of fish fitted with acoustic transmitters, provides detailed insight into movement patterns found within populations across spatial scales [23, 25]. Traditional space use studies using mark recapture techniques that physically re-capture animals are often highly resource intensive and cannot gather the same resolution of data as telemetry [22, 41]. This ability to passively and accurately track the movement of large numbers of fish allows researchers to compare individual movement patterns with life history outcomes such as growth, survival, and reproduction (e.g., [33]).

The Great Lakes Acoustic Telemetry Observation System (GLATOS) is a large scale acoustic telemetry network in the world’s largest freshwater lakes, the Laurentian Great Lakes, which allows researchers to address questions related to fish spatial ecology [26]. Walleye (*Sander vitreus*), a piscivorous top predator whose productivity is tightly tied to water temperature and clarity (preferring turbid environments between 18 and 22 °C; [9, 27], are frequently studied using the GLATOS network due to their economic importance within the Great Lakes [34], and throughout its range [27].

Within the Great Lakes, Walleye have been observed undertaking long distance migrations [18], and in some instances females travel greater distances than males [14, 35]. Access to different habitats may underlie these different movement patterns, with males preferring warmer, shallower habitats than females [30]. Further, energetics modeling indicates that lower feeding activity in male percids results in female-biased sexual size dimorphism [39]. These observations suggest that larger female Walleye may undergo migration in order to seek out higher energy prey as a strategy to achieve larger size, and therefore, fecundity [46].

**Fig. 1:**
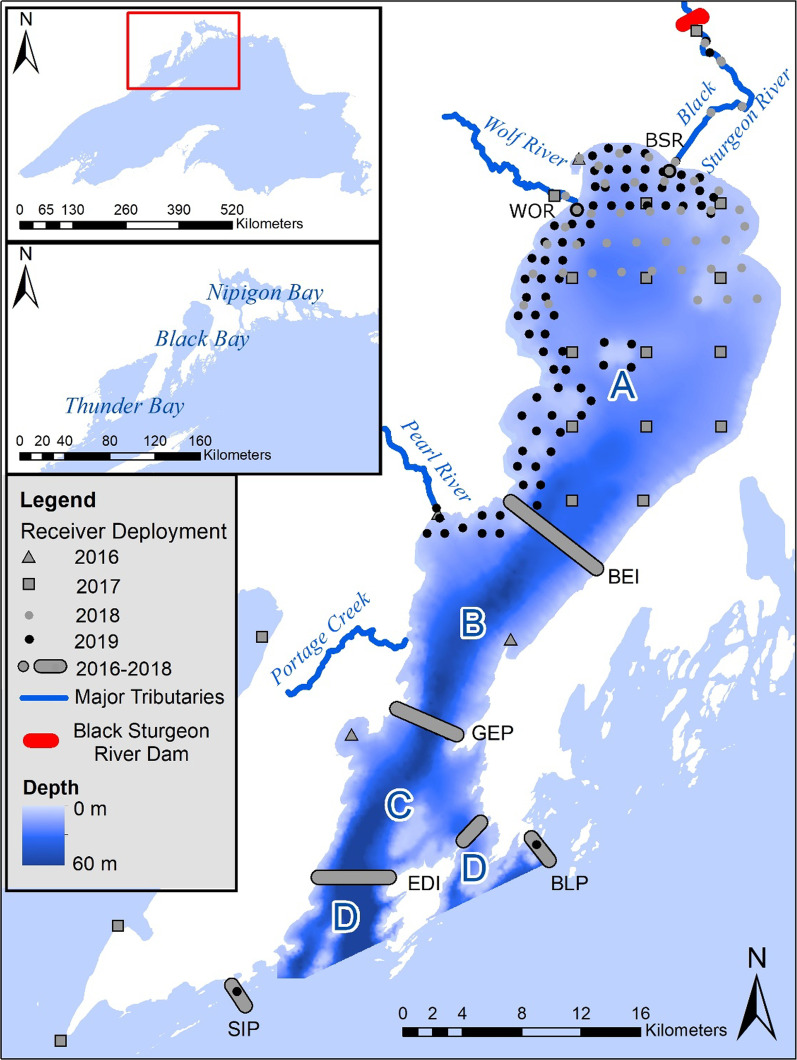
Black Bay GLATOS receiver deployment (2016–2019) with multistate mark-resight model states delineated using single letters. Grey bars denote receiver gates while circles, squares, and triangles denote individual receivers deployed during different time periods. GLATOS abbreviations: BSR-Black Sturgeon River sentinel receiver, WOR-Wolf River receivers (sentinel closest to lake—grey circle with black border), BEI-Bent Island, GEP-George Point, EDI-Edward Island, BLP-Black Bay Peninsula, SIP-Sibley Peninsula

To determine whether differential behavioural strategies are associated with life history differences in Walleye, our study makes use of a 4-year acoustic telemetry program in Black Bay, Lake Superior (Fig. 1). Black Bay is shallower, warmer, more turbid, and supports a different fish community than the deep, clear, and cold surrounding regions of Lake Superior [2]. Building on evidence elsewhere indicating differential movement patterns within Walleye populations [14, 18, 35], we aimed to first verify that this pattern of differential movement exists in Black Bay. Second, we sought to use this system of differential movement to test the POLS prediction that migratory Walleye (that leave Black Bay) have both greater maximum size (and therefore fecundity), and greater mortality than resident Walleye. Given that Walleye display sexual size dimorphism, as well as sex-based variation in mobility observed elsewhere [14, 35], we used netting data collected from Black Bay to test the hypothesis that migratory and resident Walleye life history strategies were related to sex (i.e., evidence of a greater proportion of females migrating than males). Finally, given previous research documenting the role of the environment in shaping Walleye habitat occupancy and production [9, 27], we made use of a thermal-optical habitat model to test the hypothesis that Walleye migration is driven by the amount of available optimal thermal-optical habitat within different regions of their range.

## Methods

### Fish capture and tagging

Two datasets were used in this study; Walleye movement, growth, and thermal-optical habitat use were assessed using individuals that were captured and acoustically tagged, while the spatial distributions of male and female Walleye as both juveniles and adults were assessed using data gathered in the Ontario Ministry of Natural Resources and Forestry (MNRF) Fall Walleye Index Netting (FWIN) program (see [2]).

Adult Walleye to be acoustically tagged were captured via trapnets, electrofishing, short set gill nets, and angling within and immediately outside of Black Bay, and along the lower Black Sturgeon River from 2016 to 2018 (Additional file 1: Table S1; Figure S1). Fish were measured (total length, mm), and the second dorsal spine was taken for age and growth assessment. Fish were intra-coelomically fitted with Vemco V16 (2016–2018: n = 180, random transmission intervals between 60 and 180 s) and V13 (2017: n = 8, random transmission intervals between 120 and 240 s; 2018: n = 4, random transmission intervals between 60 and 180 s) acoustic tags and released at their respective capture sites. Two experienced biologists tagged 192 Walleye in 2016 (n = 94), 2017 (n = 61), and 2018 (n = 37) following Canadian Council on Animal Care approved procedures (Lakehead University file # 1465777; Additional file 1: Supplemental Information).

### Fish tracking

The movement of acoustically tagged Walleye was tracked within and immediately outside of Black Bay using acoustic receivers. The shallower, relatively protected waters of Black Bay remain ice covered longer than the main portion of Lake Superior, with ice up typically beginning in late November and persisting until early May. The ice-free open water season of late May- early November was used to deploy, retrieve, and maintain acoustic receivers in the area. During this open water season in 2016, acoustic receivers (n = 32; Vemco VR2W, VR2AR; 69 kHz) with omnidirectional hydrophones were deployed within and outside of Black Bay to detect acoustically tagged Walleye. Receivers were suspended 0.5–1.5 m off bottom with an anchor and float system and arranged in 5 single row gates at significant ecological boundaries, with 800 m spacing between receivers (Fig. 1). These gates mark the boundary between the north and south end of Black Bay at Bent Island (BEI; n = 9), the mouth of Black Bay at George Point (GEP; n = 5), Edward Island outside of the mouth of Black Bay (EDI; n = 7), and the tips of the Sibley (SIP; n = 2) and Black Bay Peninsulas (BLP; n = 3). At each of six positions throughout the bay, individual receivers independent of gates were also deployed (hereafter referred to as sentinel receivers) to provide assessment of Walleye use at specific points of interest; two sentinel receivers were placed at the mouths of the Black Sturgeon and Wolf Rivers, where Walleye are locally known to congregate each spring, and the other sentinel receivers were placed at other points of suspected Walleye congregation throughout the bay.

During winter (November–May), receivers in water shallower than 5 m were removed to prevent damage, leaving 24 receivers. All gate receivers were re-deployed for the open water season of 2017 (May–November), as well as a grid-work of receivers in the north end of Black Bay (n = 13, 5 km spacing), and sentinel positions (n = 4; Fig. 1). All gates were redeployed again in 2018 with lower receiver coverage (BEI n = 5, GEP n = 3, EDI n = 4, SIP n = 2, BLP n = 2). In 2019, only the peninsular gates (SIP, BLP) were redeployed, each with one receiver (used for evaluating long-range movement). Data was downloaded each spring and fall, and detection efficiency was assessed at the BEI, GEP, and EDI receiver gates by simulating receiver line performance based on range testing carried out on the receivers in the study area (Additional file 1 Supplemental Information).

### Movement and survival

Walleye movement and survival was assessed from May 2016 to October 2017 using a multistate mark-resight model [6, 21] (Additional file 1: Supplemental Information) analyzed in Program MARK [48]. These years were chosen from the larger acoustic telemetry study as they preceded changes in gate deployment for other studies focusing on the inner bay not described here (Fig. 1). States that could be occupied by Walleye were designated as the areas between receiver gates (Fig. 1), and during each sighting occasion, every fish was assigned an occupied state or indicated as not sighted. The occupied state assigned to each fish for a given period was determined using a weighted average of detections of each fish at all gates and sentinel receivers at the north end of Black Bay. A transition to a new state occurred when the weighted average of detections across the gates and northern sentinel receivers fell within a new state during the next sighting occasion. This produced a condensed encounter history for each acoustically tagged Walleye describing movement throughout the study area.

To determine the appropriate time scale for evaluating patterns, movement and survival were assessed with sighting occasions at monthly and bi-weekly time intervals. Candidate models were constructed and run in Program MARK, and top explanatory models for both time intervals were selected on the basis of AIC_*c*_, where models within ΔAIC_*c*_ of 2 of the top model were considered to be equivalent [7]. The top models were used to determine time- and state-dependence on transition, survival, and sighting probabilities. Parameter estimates from the top candidate models were used to assess the probability of transitioning between states and the proportion of surviving fish in each state during each occasion.

Repeatability in individual Walleye movement patterns was assessed using the acoustic telemetry deployment from 2016 to 2018 (Fig. 1). Walleye tagged in 2016 and 2017 were assigned as either ‘migrant’ or ‘resident’ for each year that they were detected during the period when receiver gates were present (2016–2018) based on the maximum outbound extent from the northern end of Black Bay; Walleye detected beyond the George Point (GEP) receiver line (mouth of Black Bay) were considered migratory for a given year, while Walleye whose maximum outbound detection was at, or within the George Point receiver line were considered resident. In order to evaluate individual movement pattern repeatability across all 3 years of observation, generalized linear mixed models (GLMMs) framework fit with a logit link was applied. The full model we evaluated considered annual observations of Walleye migration status (stay, leave) as a function of fish length at capture (as a fixed effect) and fish ID as a random effect. Using log-likelihood ratio tests, we evaluated the significance of both the fixed and random effect comparing the top model to reduced models excluding each of these terms individually. The repeatability of individual movement patterns across years was then assessed by calculating the intra-class correlation coefficient (*R*; [32] on the model including significant variables only (see results).

Differences in age and total length between Walleye captured in 2016 with clearly defined ageing structures (n = 53) and identified as either migratory or resident were evaluated with Welch two sample *t* tests.

### Growth

Growth patterns of Walleye defined as migratory or resident in both 2016 and 2017 were determined from dorsal spine annuli by back calculating length at age using the Fraser-Lee method [5]. Only spines with clearly defined annuli were used in this analysis (n = 53). Dorsal spine annuli measurements were taken from the focus to the edge of each annulus along the horizontal elongated transect using Image J [43]. Von-Bertalanffy growth curves were fitted to both migratory and resident Walleye back calculations of length (Additional file 1: Supplemental Information). For both migratory and resident groups of Walleye, error around parameter estimates were generated using bootstrapping, drawing each size at age estimate within a group independently with replacement. Parameter estimates and associated confidence intervals were compared to determine if significant differences existed between the growth patterns of resident and migratory Black Bay Walleye.

### Habitat use

Optimal Walleye habitat area was defined as the available optimal Thermal-Optical Habitat Area (TOHA), or the benthic area where temperature and light conditions were optimal for Walleye productivity [27]. For three states within the study system (north of Bent Island, between Bent Island and George Point, and the region beyond Edward Island) monthly Secchi disk readings were taken during the 2017 open water season. Surface illuminance for 2017 was collected from the IISD Experimental Lakes Area (IISD-ELA) near Kenora, Ontario using a Kipp and Zonen SP Lite Sensor. The IISD-ELA site shares similar latitude and climate patterns to Black Bay [10], and no closer illuminance data were available. The hourly maximum and minimum depths at which optimal light conditions existed for Walleye in Black Bay were calculated as in [27] (Additional file 1: Supplemental Information). Hourly maximum and minimum optical depth preferences were averaged for each monthly period corresponding to the multistate mark-resight model to determine the depth range providing Walleye with preferred light intensities. Vertical temperature profiles were also created for the northernmost state (max depth = 14 m), the state between Bent Island and George Point (max depth = 36 m), and the southernmost state (max depth = 70 m) using temperature loggers deployed in these regions at 1 m intervals from 2 to 20 m depth, 5 m intervals from 20 to 40 m depth, and a logger at 50 m depth. Monthly temperature averages at each depth were used to determine the depth range at which conditions were optimal for Walleye (18–22 °C) [9, 27]. Depths of optimal thermal and optical habitat were then used to calculate TOHA for the three states during each monthly sighting occasion by calculating the bottom area that fell within the optimal depth ranges using ArcGIS 10.5 (Environmental Systems Research Institute, Redlands, California) using a 30 m by 30 m cell raster digital bathymetry model. The state between George Point and Edward Island was excluded from this analysis due to lost equipment, but the three states used here cover the range of habitats available to Black Bay Walleye.

Walleye habitat occupancy for each monthly sighting occasion was determined by the number of tagged fish assigned to each state in the multi-state mark-resight model. To evaluate the role of TOHA in driving Walleye occupancy, linear regressions were applied to monthly occupancy estimates within individual states, as well as a linear regression across combined data for all three states.

FWIN program data were used to assess if spatial differences existed in the relative abundance of male and female Black Bay Walleye. The relative number of male and female Walleye captured in each state of the study area were compared using Pearson’s chi-squared tests for both juvenile (0–3 years old) and adult (4 + years old) Walleye captured in FWIN nets set during September and October of 2002 to 2017. Because only one juvenile Walleye was captured in the southernmost state in any of the FWIN netting programs, this state was removed from analysis in juvenile fish to avoid violating the chi-square assumption that expected frequency of at least 80% of cells be at least five [3]. Where chi-square tests showed statistical significance, standardized residuals were calculated to assess the contribution of each cell to the significance of the model [44]. Following significant chi-squared results, the contribution of each cell (corresponding to each state for each sex) to this significance was determined by calculating cell-standardized residuals. Residuals greater than 2 indicate that the cell value is significantly higher than expected by random distribution, while residuals less than -2 indicate the cell value is significantly less than expected [44].

## Results

Walleye tagged across all years (2016–2018) were similar in age and length (Additional file 1: Table S2). Of 192 Walleye fitted with acoustic transmitters between 2016 and 2018, 184 were detected at least once on the receiver array between 2016 and 2019. The annual proportion of fish detected at each receiver gate remained consistent throughout the study period (Table 1). 179 of the tagged Walleye were detected north of the Bent Island gate at least once. Walleye were detected within the north end of Black Bay throughout the tracking period, but most of the fish congregated in this area over winter with most final fall and initial spring detections (between which north end receivers were removed) occurring north of the Bent Island each year. Walleye detections on the George Point and Edward Island receiver gates began in June, with occasional June detections as far as the peninsular gates. Walleye detections on the peninsular receiver gates (SIP, BLP) increased in July, and detections on all receivers outside of Black Bay remained highest between August and October of each year (Additional file 1: Figure S2). Between 2016 and 2019, 86 Walleye were detected beyond the George Point receiver gate. Individual movement patterns were highly variable with some fish exhibiting frequent movement back and forth between gates, while others traveled in a more linear fashion (Additional file 1: Figure S3).

**Table 1:** Annual proportion of acoustically tagged Black Bay Walleye detected on each receiver gate (relative to the total number of Walleye detected at least once in that year)

	2016 (%)	2017 (%)	2018 (%)	2019 (%)
BEI	78	75	71	NA
GEP	63	57	49	NA
EDI	45	45	41	NA
SIP	22	20	18	18
BLP	5	5	4	6

BEI, GEP, and EDI gates had reduced receiver coverage in 2018, and were not present in 2019. SIP and BLP coverage was reduced to one receiver in 2019. BEI-Bent Island, GEP-George Point, EDI-Edward Island, SIP-Sibley Peninsula, BLP-Black Bay Peninsula

**Fig. 2:**
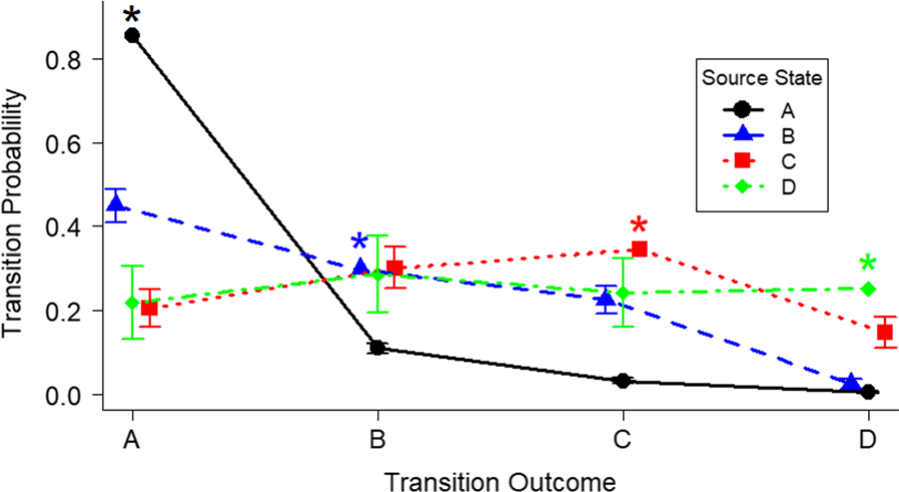
Walleye monthly transition probability between states in the Black Bay study area (A: North of Bent Island, B: Bent Island to George Point, C: George Point to Edward Island, D: Edward Island to peninsular gates; May 2016–October 2017). Standard error bars shown. *Note* Probabilities of remaining in the same state were determined from subtraction of transitions to other states from 1, and do not have error bars (marked with an asterisk). Additional missing error bars are hidden by points (source state A)

The top candidate models from the multistate mark-resight modelling for both monthly and bi-weekly sighting occasions were identical, indicating that monthly time bins captured sufficient resolution of movement in Black Bay (Additional file 1: Table S3). Models from both time periods indicated that Walleye movement was not time dependent (Additional file 1: Table S3). Transition probabilities from one state to another did, however, depend on the previous state (Fig. 2). The probability of acoustically tagged Walleye being detected was not state dependent, but varied with time, with detection probabilities remaining above 80% except for the months of October–February (Additional file 1: Figure S4). Finally, the monthly survival probability of acoustically tagged Walleye was 97.9% and was consistent across states and time (78% annual survival).

While almost all Walleye were detected in the north end of Black Bay during the winter months, two distinct sub-groups—migrators and residents—were identified within the population based on their outbound extent of migration. Log-likelihood tests indicated that both the random effect of individual ID and the fixed effect of fish length explained significantly more variation than the models from which they were absent (individual ID, *p* < 0.0001; fish length, *p* < 0.0001). Using this full model, we found significant repeatability of individual movement patterns between years, accounting for variation in fish size (*R* = 0.89, *p* < 0.0001). Migratory and resident Walleye did not differ in mean age (*t* = -0.03, *df* = 50.66, *p* = 0.97) however, total length of migratory Walleye at the time of tagging (mean = 629 ± 10.67 mm) was greater than that of residents (mean = 588 ± 9.21 mm; *t* = -2.91, *df* = 50.21, *p* = 0.005).

**Fig. 3:**
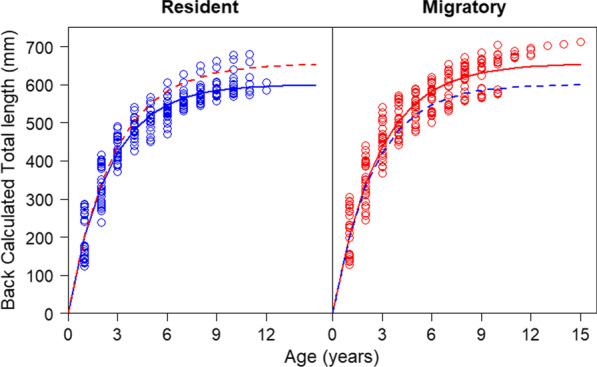
Growth trajectories of resident and migratory acoustically tagged Black Bay Walleye. Von-Bertalanffy growth curves fit to estimated length at age, back-calculated using the Fraser-Lee method (Red: Migratory Walleye, Blue: Resident Walleye; Solid lines represent growth curves of the movement strategy corresponding to the data points within the pane, dashed lines represent growth curves of opposite movement strategy for comparison)

Asymptotic lengths (*L*_∞_) differed significantly between resident and migratory Walleye based on bootstrapped 95% confidence intervals of back-calculated size at age (Migratory: *L*_∞_ = 655 mm 95% CI 642–667 mm; Resident: *L*_∞_ = 600 mm 95% CI 591–610 mm; Fig. 3). However, the curvature parameter (Brody’s *k*) of residents and migrators overlapped significantly (Migratory: *k* = 0.37 95% CI 0.34–0.39; Resident: *k* = 0.40 95% CI 0.38–0.43).

**Fig. 4:**
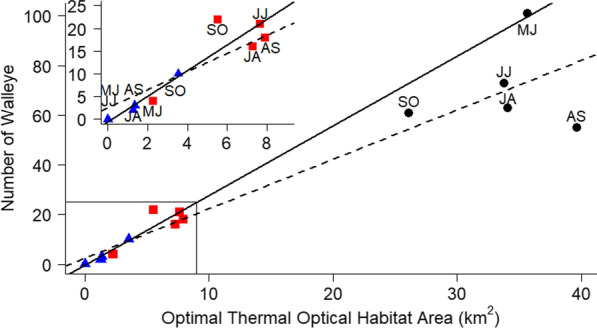
Relationship of monthly Walleye occupancy with respect to available Thermal-Optical Habitat Area in three regions of the Black Bay study system. Blue triangles represent data from the southernmost state, red squares represent data from the state between Bent Island and George Point, and black circles represent data from the northernmost state. The solid line shows the significant relationship for the southernmost state. The dashed trend line shows the relationship for all data combined. Inset shows data for the two states found within the box at the bottom left of the main plot. Monthly time periods (MJ, May 15–June 14, JJ, June 15–July 14, JA, July 15–August 14, AS, August 15–September 14, SO, September 15–October 14) of data points shown, with MJ and JJ both falling on (0,0) for the southernmost state

Walleye increased their use of habitat outside of Black Bay when the amount of available TOHA outside of the bay increased (linear regression, *F*_1,3_ = 99.26, *R*^2^ = 0.96, *p* = 0.002; Fig. 4). Although turbidity increased from the southernmost to the northernmost states, Secchi readings did not vary much over time (apart from deep measurements indicating clear water at the end of summer, Additional file 1: Figure S5), indicating that changing water temperature was the main driver of Walleye optimal habitat availability. While Walleye occupancy did not significantly vary with available TOHA in the north end of the bay (*F*_1,3_ = 0.03, *p* = 0.87), or the region between Bent Island and George Point (*F*_1,3_ = 5.10, *p* = 0.11) separately, the occupancy values fall near to those predicted by the TOHA model from outside of Black Bay (Fig. 4). A linear regression of combined occupancy and TOHA data from all states with temperature data was significant (*F*_1,13_ = 98.54, *R*^2^ = 0.87, *p* < 0.0001), but violated the assumption of homoscedasticity that log-transformation did not correct.

Adult FWIN captured Walleye showed non-random spatial distribution based on sex (*χ*^*2*^_3_ = 72.43, *p* < 0.0001), with standardized residuals indicating Walleye captured outside of Black Bay were mostly female and Walleye captured in the north end of Black Bay were mostly male (Table 2). Ratios of male:female juvenile Walleye were more even across states (*χ*^*2*^_2_ = 1.65, *p* = 0.44; Table 2). Furthermore, a larger majority of juvenile Walleye were captured in the northernmost state compared to adults.

**Table 2:** The number of adult (4 + years) and juvenile (0–3 years) male and female Walleye, and the ratio of male:female Walleye captured in each state (A: North of Bent Island, B: Bent Island to George Point, C: George Point to Edward Island, D: Edward Island to peninsular gates) by Fall Walleye Index Nets set in the Black Bay study area between the years of 2002 and 2017

State	Number of males		Number of females		Male:female
*Adult*					
A	220	*(3.34)	136	*(− 3.30)	1.62
B	33	(− 1.31)	51	(1.20)	0.65
*C*	*17*	*(− *4.01)*	*71*	*(3.96)	*0.24*
*D*	*2*	*(− *2.78)*	*21*	*(2.74)	*0.10*
*Juvenile*					
A	283		203		1.39
B	26		22		1.18
*C*	*11*		*13*		*0.85*
*D*	*1*		*0*		*NA*

Italic rows indicate areas outside of Black Bay. Standardized residuals shown in paratheses for adult fish, with an asterisk indicating statistically significant values

## Discussion

Using an acoustic telemetry network established in North America’s largest lake, we identified two distinct and repeatable Walleye movement strategies corresponding to different life history modalities. These movement strategies were highly conserved between years and split with almost 50% of the tagged population adopting either migratory or resident strategies. Consistent with the POLS hypothesis, migrant fishes achieved larger body sizes than residents. Migratory Black Bay Walleye not only achieve a larger maximum size (*L*_∞_) than residents but share a similar rate of approach to this larger maximum size (Brody’s *k*), indicating that migratory Walleye also have a faster growth rate than residents (i.e., necessary for a similar rate of approach to result in a larger maximum size). Larger body size in Walleye typically confers greater fecundity [1], with the largest or oldest females in the population having disproportionately high reproductive success [46]. Thus, fitness advantages in this migratory Walleye population may be achieved by reaching larger body sizes more quickly, conferring greater fecundity.

However, our study did not support all life history patterns predicted by PLOS. Whereas POLS predicts lower survival in migrants, multistate mark-resight modelling indicated no differences in survivorship between migrant and resident Black Bay Walleye. Because Black Bay is a fish sanctuary north of Bent Island (where all fish overwinter) and is closed throughout the bay to commercial Walleye fishing [15], this removes a major source of adult mortality. Similar survivorship between groups should lead to an increased proportion of migratory Walleye in the Black Bay population due to the fitness advantage of increased size. This, perhaps, contributed to the Walleye population growth in the region observed in the FWIN netting program [2]. While unstable environments create ideal conditions for behavioral variability predicted by POLS, long term changes to anthropogenic pressures may lead to a shift in fitness advantages of different behaviours, and eventual change in the proportion of these behaviours within a population. Humans have a long history of altering animal behaviour, including by modifying the real and perceived risks faced by animals in the face of the human “super predator” [47].

Changing climate conditions are also likely to have lasting impacts on the variability in behavioural strategies used by animals. Our findings indicate that thermal habitat conditions act as an environmental control on the timing of long-range Walleye migrations. Habitat use of migratory and resident Black Bay Walleye differed noticeably from August to October, when the water outside of Black Bay reached thermal and optical optima and Walleye occupancy outside the bay increased. Outside of late summer/early fall, water temperatures outside of Black Bay were below optimal levels for Walleye, and both migratory and resident fish shared the inner basin habitat. The apparent strong dependence of Walleye migration on thermal conditions suggests that major changes in weather or climate may greatly impact their degree of migration, such that in warmer years with longer summers (predicted to occur more frequently with climate change), migration opportunities into historically colder waters could increase. Behavioural variability expected by POLS may allow populations to adjust to changing environmental conditions however, extreme shifts associated with some climate change scenarios may result in the loss of a behavioural characteristic and lead to reduced population stability [45].

But what is the driver of annual migratory patterns in Black Bay Walleye? Given that migratory fish tend to grow larger and faster than residents, we speculate that migration is driven by access to larger-bodied, energetically-dense coregonids such as Cisco (*Coregonus artedi*), as opposed to the need to evade poor thermal-optical habitat conditions in Black Bay. Indeed, large regions inside Black Bay always fell within the optimal thermal-optical regime for Walleye between May and October, and we found no significant relationship between TOHA and Walleye occupancy within Black Bay. Access to larger, more energy dense prey has been shown elsewhere to provide increased growth efficiency and maximum size in Walleye [24]. Optimized forage intake accessed through improved food quality and larger prey size can lead to a greater energetic surplus despite increased metabolic costs to migration [38, 42]. Interestingly, the period (Aug-Oct) where migratory Walleye leave Black Bay and are able to access these different food resources coincides with the period when female Walleye increase ovary development [20].

Male and female Black Bay Walleye showed systematic sex-based space use differences in detailed Walleye netting surveys. We found that adult female Walleye were more likely to be captured outside of Black Bay than males, whereas adult male Walleye were more likely to be captured inside Black Bay than females. The timing of the netting survey coincides with the period when migratory Walleye leave Black Bay, supporting the hypothesis that females are more common among migratory fish than males. Indeed, the difference in growth exhibited between migratory and resident Walleye closely resembles the sexual size dimorphism displayed in this species [19, 39].

Notably, immature Walleye (which lack sexual growth differences) did not show the same differential spatial use pattern as sexually mature Walleye, suggesting a greater exploratory behaviour in mature (female) fish only. In previous studies examining Walleye movement, females travelled greater distances than males to access cooler, deeper water [30, 35]. Behaviours resulting in trophic niche separation have been documented in a variety of populations and communities [8, 16, 40] and one plausible explanation for female migration is that the sex benefiting most from resource acquisition (females in the case of Walleye) is the one that migrates to find those resources. Our findings indicate that hypotheses surrounding sex-biased differences in life history strategy should be considered in future work on POLS.

The tendency of mature female Walleye to be captured outside Black Bay (with no similar pattern observed in juveniles), combined with patterns in growth differences strongly suggest that migrating Walleye in our study are predominantly female. Because immature male and female Walleye grow at similar rates before maturation [39], survival should only differ for mature fish. However, despite apparent sex-based differences adult Black Bay Walleye movement patterns, survival rates appear to be similar. This may be due to the lack of significant commercial harvest of Black Bay Walleye—which was historically a major source of mortality for this population until 2003 [2, 15]—providing an equalizing effect on death rates between male/female or resident/migrant individuals.

Distinct movement patterns of migration and residency corresponded to life history differences that were largely predicted by POLS. Spatial state transitions depended on the state to which fish were previously classified, but there was no time dependence of movement patterns based on multistate mark-resight modeling. This was despite the predictable observed patterns of out-migration observed between August and October, and the congregation in the north end of the bay each winter and spring. Space use by migratory Black Bay Walleye did not differ from that of residents for much of the year, with all Walleye congregating in the north end of the bay during the winter months and spring spawning. Within both migratory and resident Black Bay Walleye groups we found variation in movement patterns; some fish made direct movements between positions, while others made frequent back and forth trips. Given our telemetry array design and study objectives, we evaluated the maximum outbound extent of movement, but not the total movement (which in highly mobile resident individuals could potentially be greater than migrants). In winter, receivers were removed from shallow regions of Black Bay to avoid ice damage, contributing to the marked decline in resight probability during this time. Detection simulations along receiver lines (which were in place over winter) indicated that a fish crossing them would be detected 99% of the time based on 2016 and 2017 deployments, suggesting Walleye movement throughout the Bay was minimal during this period and that few Walleye left the north end of Black Bay during winter.

Future work using telemetry to address questions related to animal life history will benefit by exploring multiple spatial and temporal scales. As observed in this study, variation in individual movement may occur in annual migration patterns, or in the frequency of smaller movements on the scale of hours to days. Using telemetry arrays with greater spatial resolution may reveal more exploratory vs more cautious behavioral patterns within resident populations. Greater spatial resolution in spawning areas will also allow researchers to compare behavioural differences in spawning behavior or site selection to life history outcomes such as spawning success.

## Conclusion

By combining acoustic telemetry and multistate mark-resight models with a back-calculation method of estimating fish growth, our study supports the POLS hypothesis that migratory individuals are likely to achieve larger body sizes at a given age and therefore achieve greater fitness than their resident counterparts. Further, we suspect that energetic rewards of females accessing more energy-dense prey drives this pattern of migrant fishes achieving larger maximum body size. While differences in survival were not observed, we believe that this is related to the recent removal of a major source of mortality in the form of commercial fishing. Finally, because migratory opportunities appear to be temperature-dependent, we speculate that conditions associated with climate warming may result in larger differentials in migrant vs. resident Walleye, and if these patterns are sex-dependent, increased differentials of sexual size dimorphism of Lake Superior Walleye.

## Supplementary Information

**Additional file 1**. Supplemental text and figures referenced in main text.

**Additional file 2**. R-script for determining the number of fish at each receiver gate, the number of fish detected each year, the annual migratory status of fish, and the secchi measurements of the Black Bay study system.

**Additional file 3**. R-script for back calculating the lengths of acoustically tagged Walleye.

**Additional file 4**. R-script for t-tests to assess differences in age and length of migratory and resident Walleye.

**Additional file 5**. R-script for assessing the detection efficiency of receiver gates.

**Additional file 6**. R-script for assessing the distribution of male and female Walleye captured throughout the Black Bay study system in FWIN nets.

**Additional file 7**. R-script for converting GLATOS detections into the encounter history format for Program MARK.

**Additional file 8**. R-script for assessing repeatability in Walleye migratory status using GLMMs.

**Additional file 9**. R-script for determining the TOHA available to Black Bay Walleye through the open water season.

**Additional file 10**. Migratory status (1=migratory, 0=resident) of Black Bay Walleye in 2016 (M16), 2017 (M17), and 2018 (M18) with fish length to assess the influence of length and individual on migratory status.

**Additional file 11**. Maximum outbound extent of 2016 tagged Black Bay Walleye in 2016 (G16) and 2017 (G17). 1=BEI, 2=GEP, 3=EDI, 4=BLP and SIP.

**Additional file 12**. Secchi depth measurements of the four states of the Black Bay study system through the open water season of 2017.

## Data Availability

The acoustic telemetry dataset analysed during the current study are available upon request in the GLATOS (Great Lakes Acoustic Telemetry Observation System) repository, https://glatos.glos.us/home/project/BBWAT (Date range: May 2016 to November 2019). The FWIN netting data analyzed in this study is available upon request from the Upper Great Lakes Management Unit, Ontario Ministry of Natural Resources and Forestry, Thunder Bay, Ontario, P7E6S7. Light data analyzed in this study was provided by Ken Sanilands (International Institute for Sustainable Development Experimental Lakes Area, Winnipeg, Manitoba, Canada, R3B0Y4). Other datasets and code generated and/or analyzed in the current study have been included in the Supplementary Information (Additional files 2, 3, 4, 5, 6, 7, 8, 9, 10, 11, 12).

## Abbreviations

AIC: Akaike Information Criterion
BEI: Bent Island (Receiver Gate)
BLP: Black Bay Peninsula (Receiver Gate)
EDI: Edward Island (Receiver Gate)
FWIN: Fall Walleye Index Netting
GEP: George Point (Receiver Gate)
GLATOS: Great Lakes Acoustic Telemetry Observation System
IISD-ELA: International Institute for Sustainable Development-Experimental Lakes Area
OMNRF: Ontario Ministry of Natural Resources and Forestry
POLS: Pace of Life Syndrome Hypothesis
SIP: Sibley Peninsula (Receiver Gate)
TOHA: Thermal-Optical Habitat Area

## References

[1] Baccante DA, Reid DM (1988). Fecundity changes in two exploited walleye populations. N Am J Fish Manag.

[2] Berglund E. Assessment and monitoring of Black Bay, lake superior walleye using fall walleye index netting (FWIN) 2002–2014. Upper Great Lakes Management Unit, Ontario Ministry of Natural Resources and Forestry, Thunder Bay, Ontario, 37 pp; 2014.

[3] Bewick V, Cheek L, Ball J (2004). Statistics review 8: qualitative data—tests of association. Crit Care.

[4] Biro PA, Stamps JA (2008). Are animal personality traits linked to life-history productivity?. Trends Ecol Evol.

[5] Borkholder BD, Edwards AJ (2001). Comparing the use of dorsal fin spines with scales to back-calculate length-at-age estimates in walleyes. N Am J Fish Manag.

[6] Brownie C, Hines JE, Nichols JD, Pollock KH, Hestbeck JB (1993). Capture-recapture studies for multiple strata including non-Markovian transitions. Biometrics.

[7] Burnham KP, Anderson DR. Model selection and multimodel inference: a practical information-theoretic approach (2nd ed). In: Ecological modelling, vol 172; 2002. 10.1016/j.ecolmodel.2003.11.004.

[8] Cabrol J, Trombetta T, Amaudrut S, Aulanier F, Sage R, Tremblay R, Nosais C, Starr M, Plourde S (2018). Trophic niche partitioning of dminant North-Atlantic krill species, *Meganyctiphanes norvegica*, *Thysanoesssa inermis*, and *T. raschii*. Limnol Oceanogr.

[9] Chu C, Minns CK, Moore JE, Millard ES (2004). Impact of oligotrophication, temperature, and water levels on walleye habitat in the Bay of Quinte, Lake Ontario. Trans Am Fish Soc.

[10] Columbo SJ, McKenney DW, Lawrence KM, Gray PA (2007) Climate change projections for Ontario: policymakers and planners. Applied Research and Development Branch, Ontario Ministry of Natural Resources and Forestry, Sault Ste. Marie, Ontario, 38pp.

[11] Cooke SJ, Martins EG, Struthers DP, Gutowsky LFG, Power M, Doka SE, Dettmers JM, Crook DA, Lucas MC, Holbrook CM, Krueger CC (2016). A moving target—incorporating knowledge of the spatial ecology of fish into the assessment and management of freshwater fish populations. Environ Monit Assess.

[12] Cote J, Clobert J, Brodin T, Fogarty S, Sih A (2010). Personality-dependent dispersal: characterization, ontogeny and consequences for spatially structured populations. Philos Trans R Soc B Biol Sci.

[13] Dhellemmes F, Finger J, Smukall MJ, Gruber SH, Guttridge TL, Laskowski KL, Krause J (2021). Personality-driven life history trade-offs differ in two subpopulations of free-ranging predators. J Anim Ecol.

[14] Fielder DG. Mortality, exploitation, movement, and stock size of saginaw bay walleyes, 1981–2011; 31 years of tag return analysis. Alpena Fisheries Research Station, Michigan Department of Natural Resources, Alpena, Michigan, p. 58; 2016.

[15] Furlong P, Foster RF, Colby PJ, Friday M. Black Sturgeon River dam: a barrier to the rehabilitation of Black Bay walleye. Upper Great Lakes Management Unit, Ontario Ministry of Natural Resources and Forestry; Northern Bioscience Consulting, 27pp; 2006. http://s3.documentcloud.org/documents/552802/blacksturgeonreport.pdf.

[16] Gavrilchuk K, Lesage V, Ramp C, Sears R, Bérubé M, Bearhop S, Beauplet G (2014). Trophic niche partitioning among sypatric baleen whale species following the collapse of groundfish stocks in the Northwest Atlantic. Mar Ecol Prog Ser.

[17] Harrison PM, Gutowsky LFG, Martins EG, Patterson DA, Cooke SJ, Power M (2015). Personality-dependent spatial ecology occurs independently from dispersal in wild burbot (*Lota lota*). Behav Ecol.

[18] Hayden TA, Holbrook CM, Fielder DG, Vandergoot CS, Bergstedt RA, Dettmers JM, Krueger CC, Cooke SJ (2014). Acoustic telemetry reveals large-scale migration patterns of walleye in Lake Huron. PLoS ONE.

[19] Henderson BA, Collins N, Morgan GE, Vaillancourt A (2003). Sexual size dimorphism of walleye (*Stizostedion vitreum vitreum*). Can J Fish Aquat Sci.

[20] Henderson BA, Wong JL, Nepszy SJ (1996). Reproduction of walleye in Lake Erie: allocation of energy. Can J Fish Aquat Sci.

[21] Hestbeck JB, Nichols JD, Malecki RA (1991). Estimates of movement and site fidelity using mark-resight data of wintering Canada geese. Ecology.

[22] Hussey NE, Kessel ST, Aarestrup K, Cooke SJ, Cowley PD, Fisk AT, Harcourt RG, Holland KN, Iverson SJ, Kocik JF, Mills Flemming JE, Whoriskey FG (2015). Aquatic animal telemetry: a panoramic window into the underwater world. Science.

[23] Huveneers C, Simpfendorfer CA, Kim S, Semmens JM, Hobday AJ, Pederson H, Stieglitz T, Vallee R, Webber D, Heupel MR, Peddemors V, Harcourt RG, Reynolds J (2016). The influence of environmental parameters on the performance and detection range of acoustic receivers. Methods Ecol Evol.

[24] Kaufman SD, Morgan GE, Gunn JM (2009). The role of ciscoes as prey in the trophy growth potential of walleyes. N Am J Fish Manag.

[25] Kessel ST, Cooke SJ, Heupel MR, Hussey NE, Simpfendorfer CA, Vagle S, Fisk AT (2013). A review of detection range testing in aquatic passive acoustic telemetry studies. Rev Fish Biol Fish.

[26] Krueger CC, Holbrook CM, Binder TR, Vandergroot C, Hayden TA, Hondorp DW, Nate NA, Paige K, Riley S, Fisk AT, Cooke SJ (2018). Acoustic telemetry observation systems: challenges encountered and overcome in the Laurentian Great Lakes. Can J Fish Aquat Sci.

[27] Lester NP, Dextrase AJ, Kushneriuk RS, Rawson MR, Ryan PA (2004). Light and temperature: key factors affecting walleye abundance and production. Trans Am Fish Soc.

[28] Lucas MC, Baras E (2000). Methods for studying spatial behaviour of freshwater fishes in the natural environment. Fish Fish.

[29] Luttbeg B, Sih A (2010). Risk, resources and state-dependent adaptive behavioural syndromes. Philos Trans R Soc B Biol Sci.

[30] Matley JK, Faust MD, Raby GD, Zhao Y, Robinson J, MacDougall T, Hayden TA, Fisk AT, Vandergoot CS, Krueger CC (2020). Seasonal habitat-use differences among Lake Erie’s walleye stocks. J Great Lakes Res.

[31] Montiglio PO, Dammhahn M, Dubuc Messier G, Réale D (2018). The pace-of-life syndrome revisited: the role of ecological conditions and natural history on the slow-fast continuum. Behav Ecol Sociobiol.

[32] Nakagawa S, Schielzeth H (2010). Repeatability for Gaussian and non-Gaussian data: a practical guide for biologists. Biol Rev.

[33] Nakayama S, Rapp T, Arlinghaus R (2017). Fast-slow life history is correlated with individual differences in movements and prey selection in an aquatic predator in the wild. J Anim Ecol.

[34] Poe GL, Lauber TB, Connelly NA, Creamer S, Ready RC, Stedman RC. *Net *benefits of recreational fishing in the Great Lakes Basin: a review of the literature (Issue HDRU Series No 13–10); 2013. http://www2.dnr.cornell.edu/hdru/pubs/HDRUReport13-10.pdf.

[35] Raby GD, Vandergoot CS, Hayden TA, Faust MD, Kraus RT, Dettmers JM, Cooke SJ, Zhao Y, Fisk AT, Krueger CC (2018). Does behavioural thermoregulation underlie seasonal movements in Lake Erie walleye?. Can J Fish Aquat Sci.

[36] Réale D, Dingemanse NJ, Kazem AJN, Wright J (2010). Evolutionary and ecological approaches to the study of personality. Philos Trans R Soc B Biol Sci.

[37] Réale D, Garant D, Humphries MM, Bergeron P, Careau V, Montiglio P-O (2010). Personality and the emergence of the pace-of-life syndrome concept at the population level. Philos Trans R Soc London Ser B Biol Sci.

[38] Rennie MD, Ebener MP, Wagner T (2012). Can migration mitigate the effects of ecosystem change? Patterns of dispersal, energy acquisition and allocation in Great Lakes lake whitefish (*Coregonus clupeaformis*). Adv Limnol.

[39] Rennie MD, Purchase CF, Lester N, Collins NC, Shuter BJ, Abrams PA (2008). Lazy males? Bioenergetic differences in energy acquisition and metabolism help to explain sexual size dimorphism in percids. J Anim Ecol.

[40] Robillard MM, Casselman JM, McLaughlin RL, Mackereth RW (2011). Alternative growth histories in populations of Lake Supereior brook trout: critical support for partial migration. Biol Cons.

[41] Robson DS, Regier HA (1964). Sample size in Petersen mark-recapture experiments. Trans Am Fish Soc.

[42] Roff DA (1988). The evolution of migration and some life history parameters in marine fishes. Environ Biol Fishes.

[43] Schneider CA, Rasband WS, Eliceiri KW (2012). NIH Image to ImageJ: 25 years of image analysis. Nat Methods.

[44] Sharpe D (2015). Your chi-square test is statistically significant: now what?. Pract Assess Res Eval.

[45] Shaw AK (2016). Drivers of animal migration and implications in changing environments. Evol Ecol.

[46] Venturelli P, Murphy C, Shuter B, Johnston T, van Coeverden de Groot P, Boag P, Casselman J, Montgomerie R, Wiegand M, Leggett W (2010). Maternal influences on population dynamics: evidence from an exploited freshwater fish. Ecology.

[47] Wilson MW, Ridlon AD, Gaynor KM, Gaines SD, Stier AC, Halpern BS (2020). Ecological impacts of human induced animal behaviour change. Ecol Lett.

[48] White GC, Burnham KP (1999). Program MARK: survival estimation from populations of marked animals. Bird Study.

