# Supplementary material for: Alternative migratory strategies related to life history differences in the Walleye (Sander vitreus)
Source: Mov Ecol. 2022 Mar 2;10:10. doi: 10.1186/s40462-022-00308-7 (PMC8892756; doi:10.1186/s40462-022-00308-7)
Supplement: Supplementary file 1 — Additional file 1. Supplemental text and figures referenced in main text. [file 40462_2022_308_MOESM1_ESM.docx]

**Supplemental Information**

**Text**

**Walleye Tagging Procedure**

Initial tagging procedures in 2016 used clove oil (60 mg clove oil/L water) to anesthetize Walleye, and in 2017 electric fish handling gloves (32 V-39 V, 4 mA-25 mA; Smith-Root, Vancouver, WA) were adopted. Electric fish handling glove settings were adjusted to individual fish, initially exposing the Walleye to low electrical current and voltage, and increasing both until the fish was immobilized. Anesthetized Walleye were placed in a padded trough for surgery, where a Vemco V16 (2016-2018: n=180, random transmission intervals between 60 and 180 s) or V13 (2017: n=8, random transmission intervals between 120 and 240 s; 2018: n=4, random transmission intervals between 60 and 180 s) acoustic transmitter was inserted into each fish via a small incision on the ventral side posterior to the pelvic girdle. Walleye gills were irrigated throughout the surgery process, and the incision was closed using 3 sutures (polydioxanone absorbable monofilament; Ethicon, Somerville, NJ). Walleye anesthetized using clove oil were allowed to recover in an aerated tank for 15-30 minutes prior to release, while Walleye anesthetized using electro-anesthesia gloves were released as soon as possible due to their rapid recovery time (typically less than 30 seconds).

**Simulations of Receiver Line Performance**

Detection efficiencies at the BEI, GEP, and EDI gates were tested for expanded (2016-2017) and reduced (2018) receiver coverage through detections of simulated fish. Logistic regressions were fit to range test data collected at each gate and used to characterize the probability of detection with increasing distance from a receiver in each gate. Simulated passes of 10 000 Walleye were then pushed through each receiver line at swim speeds of 1.0 m/s, which is above the maximum sustained swimming speed for Walleye found by Peake et al. (2000). Tag transmission intervals ranged from 120-360 seconds to match the minimum time delay between transmission in tags deployed in this study, and potential maximum time delay between tag transmissions in the case of misfires. Walleye detection probability for each gate was determined by the proportion of fish detected at least twice in the simulation.

Simulated detection probabilities were above 99% for each gate enclosed by land in 2016 and 2017 (BEI=99.98%, GEP=99.99%, EDI=99.95%). In 2018, with reduced gate receiver coverage simulated detection probabilities declined, but remained well above 90% (BEI=92.96%, GEP=96.21%, EDI=96.46%).

**Multistate Mark-Resight Model**

A tagged Walleye could transition from one state to another with probability ψ*_h,k_* (where *h* denotes the initial state, and *k* denotes the next location) or remain in the initial state with probability 1-ψ*_h_* where ψ*_h_*=∑ψ*_h,k_* for all possible *k*. An assumption of the multistate mark-resight model is that prior to detection in the next sighting occasion, each detected Walleye has survived the previous occasion in its initial state with probability S*_h_*. There is an additional sighting probability term that states that a Walleye may be sighted with a probability of p*_k_*. If a Walleye is not detected it may have perished (with a probability of 1-S*_h_*) or simply not been observed (with a probability of 1-p*_k_*). Each parameter of the multistate mark-resight model can be time dependent (t), state dependent (g), constant across states and time (.), or dependent on both time and state (t*g).

**Fraser-Lee Assumption of Proportional Growth Between Dorsal Spine Radius and Total Length**

The Fraser-Lee assumption of proportional growth between dorsal spine radius and total length was assessed for Black Bay Walleye by regressing length at capture against dorsal spine radius at capture with a linear model. This linear model showed a significant positive relationship between Walleye total length at capture and dorsal spine radius at capture (*L_T_* =0.19**R_c_*+371, where *L_T_*=total length at capture (mm), and *R_c_*=dorsal spine radius at capture; F1,51=36.22, R2=0.40, p<0.0001).

**Von-Bertalanffy Growth Equation**

($L=L_{\infty}(1-e^{-k\left( t-t_{0} \right)})$, where: *L*= length (mm) at age *t* (yr), *L*_∞_=asymptotic maximum length (mm), *k* is the Brody growth rate coefficient (yr^-1^), and *t*_0_= hypothetical age at which length is 0 mm). For both migratory and resident growth curves, *t_0_* was constrained to zero.

**Optimal Optical Depth Calculation for Walleye (as in Lester et al. 2004)**

The hourly maximum and minimum depths at which optimal light conditions existed for Walleye in Black Bay were calculated using the equation: $z=\frac{{-z}_{sec}}{k}\cdot{log}_{e}(\frac{I}{I_{0,t}})$, where: *z*=depth in m (calculated for both maximum and minimum preferred light intensity of Walleye), *z_sec_*=Secchi depth in m, *k* is a turbidity parameter (set to 2.1), *I*=light intensity in lux (68 lux for maximum preferred light intensity by Walleye, 8 lux for minimum preferred light intensity by Walleye), and *I_0,t_*=surface light intensity in lux for each hour.

**Tables**

Table S1. Number of Walleye fitted with acoustic transmitters from each capture location within the Black Bay system. Captures in 2016, 2017, and 2018, as well as total captures shown.

| Capture Location | Number (2016) | Number (2017) | Number (2018) | Number (Total) |
| --- | --- | --- | --- | --- |
| Black Sturgeon River | 38 | 22 | 16 | 76 |
| Hurkett Cove | 40 | 6 | 6 | 52 |
| Delany Island | 0 | 14 | 0 | 14 |
| Coldwater Creek Mouth | 0 | 19 | 15 | 34 |
| Pearl Harbour | 3 | 0 | 0 | 3 |
| Squaw Bay | 13 | 0 | 0 | 13 |

Table S2. Minimum, maximum, and mean ages and lengths of Black Bay Walleye acoustically tagged each year between 2016 and 2018.

| Year Tagged | Minimum Age (years) | Maximum Age (years) | Mean Age (years) | Minimum Length (mm) | Maximum Length (mm) | Mean Length (mm) |
| --- | --- | --- | --- | --- | --- | --- |
| 2016 | 5 | 16 | 9.6 | 471 | 782 | 611 |
| 2017 | 4 | 14 | 9.5 | 389 | 792 | 607 |
| 2018 | 4 | 15 | 9.6 | 474 | 735 | 614 |

Table S3. Top 5 candidate models for monthly and bi-weekly sighting occasions between May 2016 and September 2017 describing the effects of time dependence (t), state dependence (g), time and state dependence (t*g), and no time or state dependence (.) on transition probabilities (ψ), sighting probabilities (p) and survival probabilities (S) in acoustically tagged Black Bay Walleye. AIC_c_, ΔAIC_c_, AIC_c_ weight, model likelihood, number of parameters and deviance shown.

|  | Model | AIC_c_ | ΔAIC_c_ | AIC_c_ Weight | Model Likelihood | Number of Parameters | Deviance |
| --- | --- | --- | --- | --- | --- | --- | --- |
| *Monthly* |  |  |  |  |  |  |  |
|  | ψ(g)p(t)S(.) | 2407.8557 | 0.0000 | 0.93207 | 1.0000 | 30 | 1619.2802 |
|  | ψ(g)p(t)S(t) | 2413.9037 | 5.2380 | 0.06793 | 0.0729 | 45 | 1663.9335 |
|  | ψ(g)p(t*g)S(.) | 2433.9134 | 26.0577 | 0.0000 | 0.0000 | 81 | 1601.9016 |
|  | ψ(g)p(t*g)S(g) | 2434.4543 | 26.5986 | 0.0000 | 0.0000 | 84 | 1595.2231 |
|  | ψ(g)p(t*g)S(t) | 2442.6430 | 34.7873 | 0.0000 | 0.0000 | 97 | 1571.5340 |
| *Bi-weekly* |  |  |  |  |  |  |  |
|  | ψ(g)p(t)S(.) | 4862.4568 | 0.0000 | 0.86896 | 1.0000 | 50 | 4163.5488 |
|  | ψ(g)p(t)S(g) | 4866.2404 | 3.7836 | 0.13104 | 0.1508 | 53 | 4160.9546 |
|  | ψ(g)p(t*g)S(t) | 4972.8223 | 65.3655 | 0.0000 | 0.0000 | 197 | 3887.0301 |
|  | ψ(.)p(t*g)S(.) | 5059.4471 | 196.9903 | 0.0000 | 0.0000 | 3 | 4457.5627 |
|  | ψ(g)p(t*g)S(t*g) | 5186.1336 | 323.6768 | 0.0000 | 0.0000 | 307 | 3843.2729 |

**Figures**


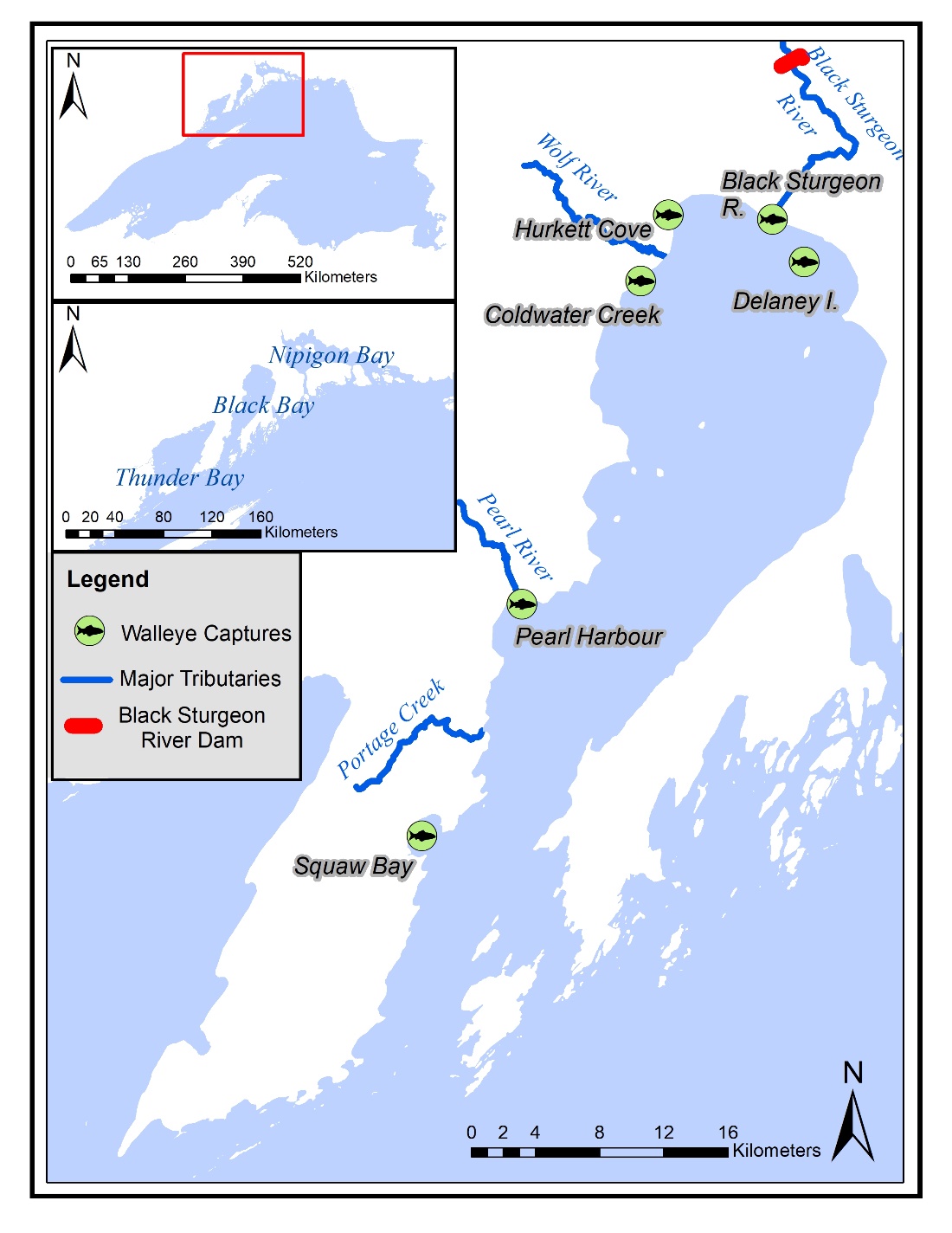


Figure S1. Capture locations for Walleye captured from the Black Bay study system and fitted with acoustic transmitters (2016-2018).


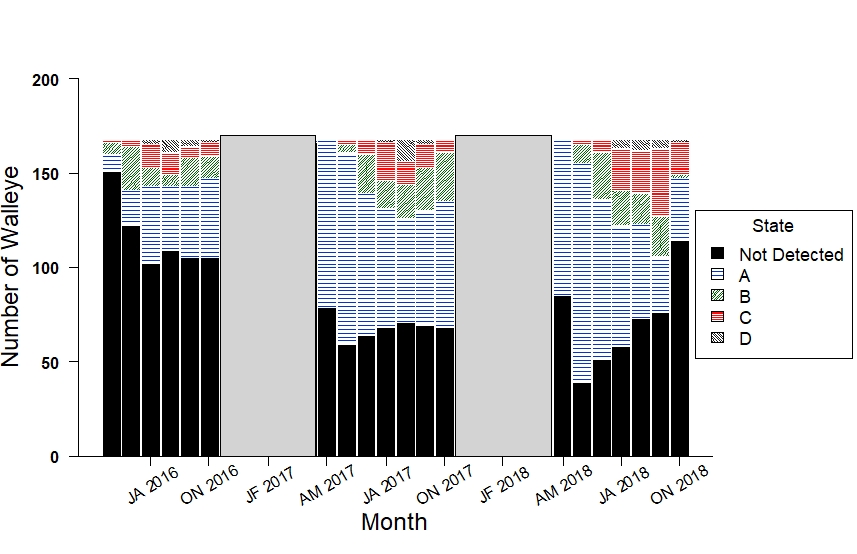
Figure S2. The number of acoustically tagged Black Bay Walleye detected in each state of the Black Bay study system during each monthly occasion from 2016-2018. Grey boxes indicate periods with reduced receiver coverage. (Abbreviations represent time periods during the study: AM, April 15-May 14, JA, July 15-August 14, ON, October 15-November 14).


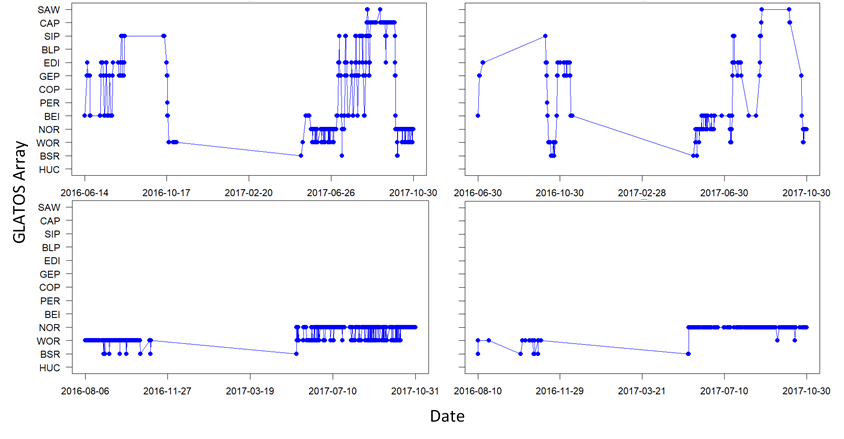


A

B

C

D

Figure S3. Abacus plots of individual Walleye fitted with acoustic transmitters showing detections at GLATOS Arrays (HUC-Hurkett Cove, BSR-Black Sturgeon River, WOR-Wolf River, NOR-North Grid, BEI-Bent Island, PER-Pearl River, COP-Copper Point, GEP-George Point, EDI-Edward Island, BLP-Black Bay Peninsula, SIP-Sibley Peninsula, CAP-Thunder Cape, SAW-Sawyer Bay. Plots shown for A) high frequency movements by a migratory fish, B) direct movements by a migratory fish, C) high frequency movements by a resident fish, and D) direct movements by a resident fish.


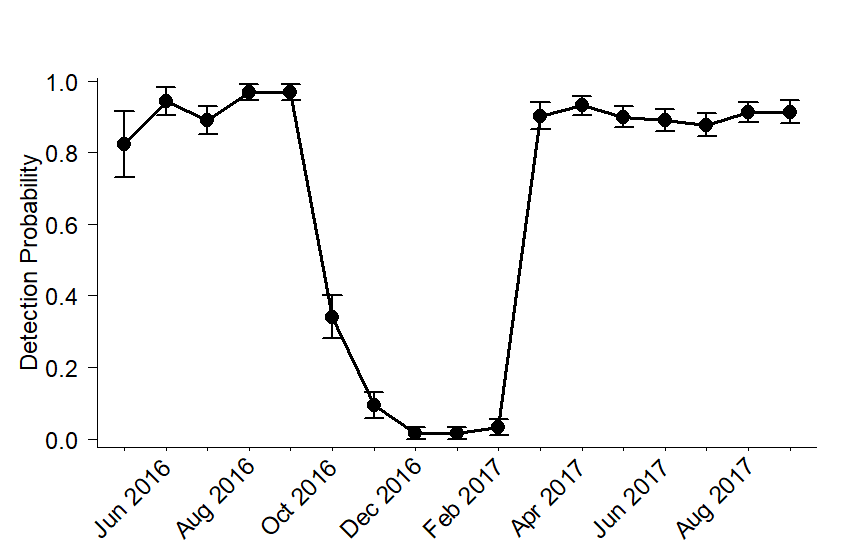


Figure S4. Monthly detection probabilities with standard error bars assessed in a multistate mark-resight model for acoustically tagged Walleye throughout the Black Bay study area (May 2016-Sept 2017).


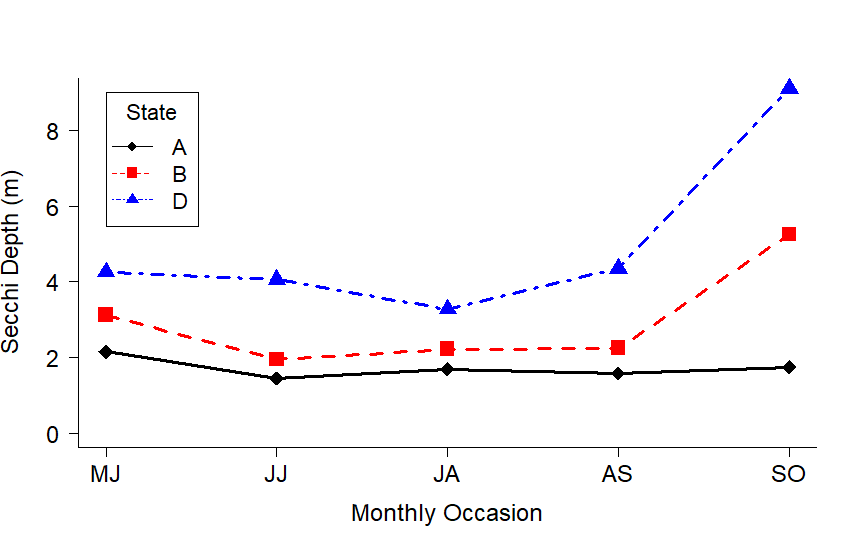
Figure S5. Secchi disk depth measurement for each monthly occasion in each state of the Black Bay study area. (Abbreviations represent time periods during the study: MJ, May 15-June 14, JJ, June 15-July 14, JA, July 15-August 14, AS, August 15-September 14, SO, September 15-October 14).
